# The role of longevity-related genetic variant interactions as predictors of survival after 85 years of age

**DOI:** 10.1016/j.mad.2024.111926

**Published:** 2024-06

**Authors:** Maja Šetinc, Željka Celinšćak, Luka Bočkor, Matea Zajc Petranović, Anita Stojanović Marković, Marijana Peričić Salihović, Joris Deelen, Tatjana Škarić-Jurić

**Affiliations:** a Institute for Anthropological Research, Zagreb 10000, Croatia; b Centre for Applied Bioanthropology, Institute for Anthropological Research, Zagreb 10000, Croatia; c Max Planck Institute for Biology of Ageing, Cologne 50931, Germany; d Cologne Excellence Cluster on Cellular Stress Responses in Ageing-Associated Diseases (CECAD), University of Cologne, Cologne 50931, Germany

**Keywords:** Longevity, Genetics, Survival, SNP interaction, Epistasis, Health-related traits

## Abstract

Genome-wide association studies and candidate gene studies have identified several genetic variants that might play a role in achieving longevity. This study investigates interactions between pairs of those single nucleotide polymorphisms (SNPs) and their effect on survival above the age of 85 in a sample of 327 Croatian individuals. Although none of the SNPs individually showed a significant effect on survival in this sample, 14 of the 359 interactions tested (between SNPs not in LD) reached the level of nominal significance (p<0.05), showing a potential effect on late-life survival. Notably, *SH2B3* rs3184504 interacted with different SNPs near *TERC*, *TP53* rs1042522 with different SNPs located near the *CDKN2B* gene, and *CDKN2B* rs1333049 with different SNPs in *FOXO3*, as well as with *LINC02227* rs2149954. The other interaction pairs with a possible effect on survival were *FOXO3* rs2802292 and *ERCC2* rs50871, *IL6* rs1800795 and *GHRHR* rs2267723, *LINC02227* rs2149954 and *PARK7* rs225119, as well as *PARK7* rs225119 and *PTPN1* rs6067484. These interactions remained significant when tested together with a set of health-related variables that also had a significant effect on survival above 85 years. In conclusion, our results confirm the central role of genetic regulation of insulin signalling and cell cycle control in longevity.

## Highlights

• Genetic interactions that possibly impact survival in old age (85+ years).
• Interactions between longevity-associated variants tested for survival >85 years.
• Several interactions with a putative effect on survival >85 years were identified.
• The genetic interactions affect survival independent of health-related parameters.
• Interacting SNPs involved in insulin signaling, cell cycle control and DNA repair.

## 1 Introduction

Ageing is a complex process of organismal changes influenced by environmental factors and modulated by a complex system of gene regulation. It is defined by progressive weakening of all the functions of the organism, which ultimately leads to its death (Kirkwood, 2005). Since the world is facing ageing of the global population, with the proportion of elderly expected to almost double by 2050 (World Health Organization, 2023), the importance of research on this topic has never been greater. Most basic ageing mechanisms and candidate genes that affect them were discovered in model organisms (Antebi, 2007), but many retain the same function in humans due to high conservation of those genes among species (Smulders and Deelen, 2023). Research into these mechanisms is essential for a better understanding of what drives the ageing process, as well as for discovering the factors that contribute to successful ageing and longevity. Also, studies of the complex cellular signalling network that regulates the ageing process indicate its plasticity (Campisi et al., 2019) and point to ways in which it can be influenced.

The connection between food intake and lifespan has long been established (Fontana et al., 2010), and with it the involvement of the insulin/insulin-like growth factor signalling pathway (IIS). The key role of the IIS in ageing is exemplified by studies of mice carrying mutations in key genes downstream of insulin receptors (such as IRS1 (Selman et al., 2008)), as well as drugs that modulate insulin sensitivity or boost autophagy (Curtis et al., 2005, Rubinsztein et al., 2011). However, it is likely that many other pathways also play a role. Accumulation of DNA damage and telomere shortening are both time-related processes that accompany ageing (Vijg, 2000), and the mechanisms that affect DNA repair and control cell cycle progression are key for maintaining genomic integrity as an organism ages (Lombard et al., 2005). Age is a major risk factor for developing age-related chronic conditions (Niccoli and Partridge, 2012, Dillin et al., 2014, Hou et al., 2019), which is why genes modulating the risk for chronic disease have also been studied as candidate genes for longevity. The most well-known example is the *APOE* gene – associated with the risk for cardiovascular diseases (Eichner et al., 1993, Wilson et al., 1994, Bennet et al., 2007) and Alzheimer’s disease (Zuo et al., 2006, Farrer et al., 1997) – which was first identified in candidate gene studies and later confirmed in genome-wide association studies (GWAS) as the most important genetic factor influencing longevity (Smulders and Deelen, 2023).

Longevity is a complex trait, shaped both by the environment and genetic background, as well as by interactions between different genes involved in various signalling pathways (Shadyab and LaCroix, 2015, Brooks-Wilson, 2013). As GWAS focus on identifying the effects of individual SNPs (Lin et al., 2017), the insight gained from these studies is often fragmentary and does not consider the way these genes interact with each other or act in regard to a broader genetic context. When the complexity of the ageing process is considered, it is clear that gene-gene interactions, or epistasis, should also be explored, as complex interactions may be more important than the independent main effects of any one susceptibility gene (Moore, 2003). Analysing statistical interaction between loci can both increase the power to detect effects as well as outline the biological and biochemical pathways that underpin the phenotype (Cordell, 2009). This approach has been successfully used by Dato et al. (2018), who looked at interactions between SNPs belonging to three candidate pathways – IIS, DNA repair and pro/antioxidant pathways – to determine the combined effect of these SNPs on longevity, thus proving the validity of this approach for studying the genetics of ageing (Dato et al., 2018).

In this study, we tested the effect of 43 SNPs, previously reported to have an effect on longevity and associated with genes belonging to different ageing-related pathways, on survival of the oldest-olds, both individually and in SNP-SNP interactions. To this end, we made use of our previously generated dataset on a Croatian sample of individuals aged 85 years and older, which has been used to determine the genetic makeup that contributes to reaching longevity and extreme longevity in the studied sample (Šetinc et al., 2023). Furthermore, the significance of these interactions was tested together with a large set of health status indicators available for the studied population to determine whether the genetic effect was independent of health-related phenotypes.

## 2 Materials and methods

### 2.1 Study population

The study sample consisted 327 unrelated oldest-old adults (85 years and older) who were residents of one of the 13 homes for elderly and infirm in Zagreb area (Croatia) in the period between 2007 and 2009 when the field research was carried out. Each subject participated voluntarily, signing an informed consent for participation and an additional consent for providing a peripheral venous blood sample for biochemical, haematological, and genetic analyses. Biochemical and haematological parameters were determined in an accredited laboratory. All subjects were interviewed, a short anthropometry was performed, their blood pressure was measured, and an ultrasound densitometry of the calcaneus (heel bone) was performed using Sahara Bone Densitometer (Hologic, Marlborough, Massachusetts, United States). The comprehensive questionnaire used in the research contained a wide spectrum of questions about functional ability, quality of life, family history of health and longevity, health and health-related behaviours, as well as two internationally standardised questionnaires: Mini Nutritional Assessment (MNA) for assessing nutritional status (Guigoz and Vellas, 1999) and the psychometric test Mini Mental State Examination (MMSE) for assessing the mental state of respondents (Folstein et al., 1975). A detailed description of the sample and study protocol can be found in Perinić Lewis et al. (2022) (Perinić Lewis et al., 2022). Ten years after the initial survey, the date of death for each of the respondents was collected from the national mortality register. Peripheral blood samples of 100 unrelated young people between the ages of 20 and 35 were collected (using the snowball method, with the aim of collecting a sample of individuals with random chances for reaching advanced old age) as a reference group for calculating the relative telomere length of the older adult subjects. The only inclusion criteria for this group were Croatian citizenship (in second generation) and the year of birth, but additional care was taken to make sure that sex distribution and age variance of the control group of young individuals aligned to that of the elderly sample.

The sample collection and the research described here were approved by the Ethics Committee of Institute for Anthropological Research (Zagreb, Croatia) and performed following all institutional guidelines. Ethical approvals obtained on March 4th 2006 (130–981/06) and 22nd November 2018 (20180518).

### 2.2 DNA isolation and genotyping

DNA was isolated from peripheral blood using the salting-out method (Miller et al., 1988). Forty-three SNPs located in candidate longevity genes were selected by reviewing the relevant literature, with the main criteria for inclusion being a strong or repeated association with human longevity and involvement in various signalling and metabolic pathways that play a role in the ageing process (e.g., cell cycle regulation, DNA repair mechanisms, the IIS). The DNA samples of all subjects were genotyped in a commercial laboratory using Kompetitive Allele Specific Polymerase chain reaction (KASP). Out of the initial 327 subjects from the elderly group, genotyping was unsuccessful for 13 subjects at nine or more loci (over 20% of data was missing) and they were therefore excluded from further analyses, leaving a final sample of 314 participants. All missing data for participants with 1–8 unsuccessfully genotyped SNPs were replaced by the median value for that SNP.

### 2.3 Measurement of relative telomere length

Relative telomere length (RTL) was measured by quantitative polymerase chain reaction (qPCR) using primers that specifically bind to telomeric repeats (Cawthon, 2002). To calculate the relative telomere length of each subject, two reactions are needed: one in which specific primers multiply telomeric repeats, and another in which a gene that is repeated only once in the human genome is multiplied (in this case, the gene for beta-globin was chosen). We used 200 nM of following primers: *tel1* [5′-CGGTTT(GTTTGG)_5_GTT-3′] and *tel2* [5′-GGCTTG(CCTTAC)_5_CCT-3′] for the telomere repeats, and *hbg1* [5′GCTTCTGACACAACTGTGTTCACTAGC-3′] and *hbg2* [5′-CACCAACTTCATCCACGTTCACC-3′] for single-copy gene human beta-globin, as they were listed in the protocol by Lin et al. (2010) (Lin et al., 2010) adapted from Cawthon (2002) (Cawthon, 2002). We used Brilliant III Ultra-Fast SYBR® Green QPCR Master Mix with Low ROX (Agilent Biotechnologies, Santa Clara, California, United States) and added 50 ng of DNA per reaction, which was run on the Agilent AriaMX Real-time PCR System. The thermal cycling profile consisted of: 2 min preheating at 50 °C, 2 min denaturation of the samples at 96 °C, followed by 35 cycles of denaturation at 96 °C lasting 15 s and annealing/extension at 54 °C for 60 s. All samples were run in triplicates. We performed qPCR for both the 85+ sample we wanted to determine the relative telomere length for, and a control group of young people that was used as a reference sample. The relative telomere length was then expressed by fold change which represents the difference between the ratio of multiplied telomeric DNA and reference gene DNA of the target sample compared to the reference sample (Cawthon, 2002), according to the following formula: 2 ^–(∆Ct(old) – mean ∆Ct(young)^ = 2 ^–ΔΔCt^. The fold change calculated in this manner is proportional to the average length of telomeres in the subjects' leukocytes, and the obtained data was used as a variable in further analyses.

### 2.4 Statistical analyses

Genotype data (available in open access on the online repository Zenodo) (Šetinc et al., 2022) were coded as follows: homozygotes were given a value of 0 or 2, and heterozygotes were assigned a value of 1. The value of 2 was given to the allele that has been associated with increased longevity in previous research (Supplementary Table 1). The participants whose exact date of death was unknown were censored, and the target variable for calculating survival was set as the number of years the participants had lived after the age of 85. First, a Cox regression analysis testing the effect of each SNP on survival above 85 years was performed, with bootstrapping using 1000 samples and correction for gender. In order to avoid false-positive results caused by an extremely small representation of a single genotype, all SNPs with less than 10 cases of homozygous genotypes of either type were excluded from further analyses (n = 15). The remaining SNPs were tested for LD using Haploview (Barrett et al., 2005), and all possible SNP-SNP interactions between two SNPs that were not in LD (r < 0.2) were tested in survival analysis (359 interactions in total, listed in Supplementary Table 2). The effect of the SNP-SNP interaction on survival was tested using a bootstrapped Cox regression model that included gender, both of the SNPs (to account for their individual effect on the model), and their interaction as the variables. Survival analysis was also performed for RTL, which was tested both univariately and as a part of the health-related dataset. The subset of variables out of this health-related dataset that had a significant effect on survival were tested once again using Cox regression analysis with bootstrapping, and the ones that reached statistical significance were added to the regression models with significant SNP-SNP interactions. All statistical analyses were performed using SPSS software package 21.0.

## 3 Results

### 3.1 Single SNP and interaction analyses

We first tested each of the 43 genotyped SNPs to determine their individual effect on survival after 85 years of age. However, none of the SNPs showed a significant effect on survival in our sample (Table 1). Relative telomere length, when tested univariately, was also not a significant predictor of survival.

**Table 1:** The results of the Cox regression survival analysis for each of 43 longevity SNPs in the Croatian oldest-old sample.

**SNP**	**Variant type**	**Associated gene**	**Gene most likely impacted**	**Cox regression**				**included in SNP-SNP analysis**
				**p-value**	**Hazard Ratio (HR)**	**95% CI for HR**		
						**Lower**	**Upper**	
rs225119	intronic	*PARK7*	*PARK7*	0.626	0.956	0.795	1.142	*
rs2360675	intronic	*KLF7*	*KLF7*	0.740	0.969	0.811	1.151	*
rs12696304	regulatory region variant	*TERC*	*ACTRT3*	0.257	0.897	0.731	1.082	*
rs3772190	intronic	*TERC*	*ACTRT3*	0.095	1.168	0.974	1.408	*
rs16847897	intronic	*TERC*	*ACTRT3*	0.078	0.849	0.705	1.011	*
rs572169	synonymous	*GHSR*	*GHSR*	0.746	1.030	0.845	1.228	*
rs33954691	synonymous	*TERT*	*TERT*	0.928	1.013	0.809	1.297	
rs2706372	intronic	*RAD50/IL13*	*IL13*	0.985	1.002	0.831	1.234	*
rs2149954	intronic	*LINC02227*	*(no data)*	0.464	0.942	0.801	1.114	*
rs12203592	intronic	*IRF4*	*IRF4*	0.059	0.763	0.568	1.016	
rs1800629	regulatory region variant	*TNF*	*HLA-C*	0.622	0.930	0.689	1.212	
rs12206094	intronic	*FOXO3*	*FOXO3*	0.804	1.021	0.870	1.221	*
rs2802292	intronic	*FOXO3*	*FOXO3*	0.487	0.938	0.791	1.122	*
rs2764264	intronic	*FOXO3*	*FOXO3*	0.708	0.969	0.824	1.156	*
rs10457180	intronic	*FOXO3*	*FOXO3*	0.751	0.973	0.815	1.158	*
rs13217795	intronic	*FOXO3*	*FOXO3*	0.603	0.955	0.810	1.133	*
rs4946935	intronic	*FOXO3*	*FOXO3*	0.729	1.031	0.879	1.232	*
rs9456497	intronic	*IGF2R*	*IGF2R*	0.749	0.968	0.773	1.189	*
rs10455872	intronic	*LPA*	*SLC22A3*	0.711	0.919	0.596	1.445	
rs1800795	intronic	*IL6*	*STEAP1B*	0.714	0.971	0.823	1.149	*
rs2069837	non-coding exon variant	*IL6*	*IL6*	0.427	1.128	0.841	1.501	
rs2267723	intronic	*GHRHR*	*GHRHR*	0.681	0.968	0.817	1.133	*
rs13251813	intronic	*WRN*	*WRN*	0.587	1.120	0.741	1.747	
rs4977756	intronic	*CDKN2B*	*CDKN2B*	0.835	1.018	0.851	1.215	*
rs1333049	intronic	*CDKN2B*	*CDKN2B*	0.642	1.037	0.891	1.221	*
rs4837525	intronic	*PAPPA*	*PAPPA*	0.162	0.883	0.751	1.049	*
rs533984	intronic	*MRE11A*	*MRE11A*	0.491	1.064	0.892	1.273	*
rs17202060	intronic	*TXNRD1*	*TXNRD1*	0.407	1.076	0.900	1.309	*
rs3184504	missense	*SH2B3*	*SH2B3*	0.357	0.921	0.780	1.102	*
rs1207362	intronic	*KLOTHO*	*KLOTHO*	0.269	0.911	0.767	1.066	*
rs9536314	missense	*KLOTHO*	*KLOTHO*	0.136	1.200	0.924	1.554	
rs9527025	missense	*KLOTHO*	*KLOTHO*	0.171	0.833	0.649	1.106	
rs2229765	missense	*IGF1R*	*IGF1R*	0.142	1.114	0.955	1.275	*
rs12437963	intronic	*IGF1R*	*IGF1R*	0.791	0.970	0.769	1.213	
rs1042522	missense	*TP53*	*TP53*	0.133	0.870	0.714	1.048	*
rs2078486	intronic	*TP53*	*EFNB3*	0.550	0.899	0.644	1.290	
rs107251	intronic	*SIRT6*	*SIRT6*	0.984	1.004	0.718	1.320	
rs2075650	intronic	*TOMM40*	*TOMM40*	0.709	0.949	0.705	1.225	
rs429358	missense	*APOE*	*APOE*	0.106	0.784	0.556	1.045	
rs7412	missense	*APOE*	*APOE*	0.345	0.879	0.676	1.153	
rs4420638	regulatory region variant	*APOC1*	*APOE*	0.262	0.871	0.668	1.129	
rs50871	intronic	*ERCC2*	*KLC3*	0.654	0.967	0.824	1.127	*
rs6067484	intronic	*PTPN1*	*PTPN1*	0.241	0.894	0.737	1.099	*

In order, the columns show rsID of tested SNPs, variant type, gene (both the gene that has been associated with the SNP in other publications and the gene reported to most likely be affected by the SNP by eQTL or Variant2Gene pipeline in the online database Open Targets Genetics), bootstrap-adjusted p-values, hazard ratios (HR) and adjusted 95% confidence intervals (CI) for HR obtained in a Cox regression analysis of single SNP and gender with survival time after 85 years of age as the time-to-event variable. SNPs that pass the criterion of having over 10 cases of any genotype represented in our sample and have been included in further analyses are marked with an asterisk.

**Fig. 1:**
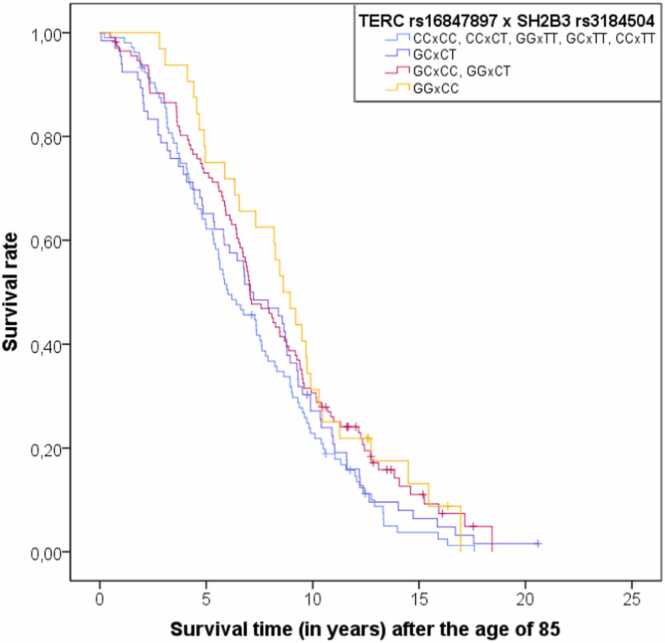
Kaplan-Meier curve of survival after the age of 85 for the interaction between *TERC* rs16847897 and *SH2B3* rs3184504 (p=0.002). Nine possible genotype combinations are grouped in four categories (4, 2, 1, 0) based on the product value of the genotype scores. The genotype combination with a value of four (marked in orange) has two longevity-associated effect alleles on each locus; the combinations with a value of two (magenta) have two longevity-associated effect alleles on one locus and one on the other; heterozygous genotype combination with the value of one (indigo) have one longevity-associated effect allele on each locus, and the genotypes with a value of zero (blue) have no longevity-associated effect alleles on at least one of the two loci.

As a next step, we studied the interactions between the SNPs. Out of the 359 tested models (Supplementary Table 2), 14 different SNP combinations showed a bootstrap-adjusted nominally significant interaction effect on survival after 85 years (Table 2). Out of 14 interactions, nine are combinations between three gene pairs – *TERC* and *SH2B3*, *TP53* and *CDKN2B*, and *CDKN2B* and *FOXO3*. Missense variant rs3184504 in *SH2B3* had an effect on late-life survival in interactions with three intronic SNPs located near *TERC*: rs16847897 (p=0.002), rs12696304 (p=0.014) and rs3772190 (p=0.032). A missense mutation in *TP53*, rs1042522, made significant interaction pairs with two intronic SNPs located near *CDKN2B*, rs4977756 (p=0.003) and rs1333049 (p=0.025). The variant rs1333049 near *CDKN2B* also impacted survival above 85 years in separate interactions with intronic *FOXO3* SNPs rs4946935 (p=0.009), rs12206094 (p=0.021)*,* rs13217795 (p=0.043) and rs2764264 (p=0.049). Multiple interactions between a single SNP and variants located in close genomic proximity corroborate the finding that these genes in tandem could affect survival, even though their repeated pairing could also be due to the high LD between the *TERC*, *CDKN2B* and *FOXO3* variants, respectively. Other interaction pairs that affected survival above 85 years were *FOXO3* rs2802292 and *ERCC2* rs50871 (p=0.013), *CDKN2B* rs1333049 and *LINC02227* rs2149954 (p=0.038), *IL6* rs1800795 and *GHRHR* rs2267723 (p=0.038), *LINC02227* rs2149954 and *PARK7* rs225119 (p=0.044), and *PARK7* rs225119 and *PTPN1* rs6067484 (p=0.045). We applied a false discovery rate (FDR) correction to the interaction p-values, but none of the SNP-SNP interactions passed this threshold (p<1.39 ×10^−4^). Fig. 1 depicts the Kaplan-Meier curve of survival after the age of 85 for the interaction between *TERC* rs16847897 and *SH2B3* rs3184504 (p=0.002, our strongest finding), which shows how different genotype combinations impact the late-life survival. It is visible that the respondents who are carriers of homozygous genotypes associated with longevity for both SNPs in interaction have better survival than carriers of the other genotype combinations.

**Table 2:** The results of Cox regression analysis of SNP-SNP interactions as predictors of survival above 85 years in a Croatian sample.

First SNP	Second SNP	Interaction p-value	Hazard Ratio (HR)	95% CI for HR	
				Lower	Upper
***TERC*****rs16847897**	***SH2B3*****rs3184504**	**0.002**	0.665	0.512	0.860
***CDKN2B*****rs4977756**	***TP53*****rs1042522**	**0.003**	1.512	1.135	2.119
***FOXO3*****rs4946935**	***CDKN2B*****rs1333049**	**0.009**	1.306	1.066	1.654
***FOXO3*****rs2802292**	***ERCC2*****rs50871**	**0.013**	0.750	0.584	0.940
***TERC*****rs12696304**	***SH2B3*****rs3184504**	**0.014**	0.708	0.539	0.946
***FOXO3*****rs12206094**	***CDKN2B*****rs1333049**	**0.021**	1.292	1.042	1.642
***CDKN2B*****rs1333049**	***TP53*****rs1042522**	**0.025**	1.336	1.030	1.738
***TERC*****rs3772190**	***SH2B3*****rs3184504**	**0.032**	1.403	1.007	1.927
***LINC02227*****rs2149954**	***CDKN2B*****rs1333049**	**0.038**	0.785	0.619	0.983
***IL6*****rs1800795**	***GHRHR*****rs2267723**	**0.038**	1.246	1.000	1.537
***FOXO3*****rs13217795**	***CDKN2B*****rs1333049**	**0.043**	1.279	1.021	1.649
***PARK7*****rs225119**	***LINC02227*****rs2149954**	**0.044**	0.776	0.605	1.002
***PARK7*****rs225119**	***PTPN1*****rs6067484**	**0.045**	1.366	0.986	1.883
***FOXO3*****rs2764264**	***CDKN2B*****rs1333049**	**0.049**	1.266	1.005	1.624

Each model included gender, two SNPs and their interaction as predictor variables. Presented in the table are the bootstrap-adjusted p-values from regression models of all significant SNP-SNP interactions, as well as interaction HR and adjusted 95% CI for HR. Significant p-values are marked in bold.

### 3.2 Health-related measures contributing to survival

Given the large amount of health data collected from the participants, we created a comprehensive set of 33 variables covering a large spectrum of health-related parameters and then used Cox regression to determine which of those factors also contribute to the survival of our oldest-old sample (Table 3). In this model, nine out of the 33 health-related variables tested simultaneously remained significantly associated with survival in advanced old age. Higher odds of surviving past 85 were found for participants who had a family history of longevity, with either a mother or a sibling living beyond 80 years of age. Moreover, participants who were categorised as well-nourished and fell among the first three quartiles of the weight distribution had higher chances of survival as well. In addition, higher chances of survival were found among those who reported taking less than four medicaments daily and taking B-complex supplements. Folate levels above 18,1 nmol/L and one or less hospital stays in the year prior to taking the survey were also significantly associated with higher chances of survival. Interestingly, higher chances of survival in advanced old age were found for participants who had osteopenia (lowered bone density), but not osteoporosis. When these nine variables were tested in a separate model (Supplementary Table 3), all of them remained significant. RTL, which was also tested jointly with the health-related variables, did not have a significant effect on survival chances of the studied sample.

**Table 3:** Cox regression analysis of gender, 33 health-related variables and relative telomere length (RTL) as predictors of survival above 85 years of age, performed with bootstrapping using 1000 samples.

**Predictor variables (referent values)**	**According to beta value, longer survival with following characteristics**	**p-value**	**Hazard Ratio (HR)**	**95% CI for HR**	
				**Lower**	**Upper**
**Gender (men)**	Women	0.546	0.869	0.489	1.390
**Body weight by sex-specific 4**^**th**^**quartile (men = 87.3+ kg; women = 72.6+ kg)**	Body weight: men = <87.3 kg; women = <72.6 kg	**0.014**	0.576	0.340	0.949
**Waist circumference by median (men = 100.0+ cm; women = 92.0+)**	Waist circumference: men = 100.0+ cm; women = 92.0+	0.110	1.352	0.902	2.036
**Upper arm circumference by median (men = 27.6+ cm; women = 27.3+ cm)**	Upper arm circumference: men = < 27.6 cm; women = <27.3 cm	0.167	0.741	0.441	1.102
**Left heel bone mineral density (T-values: > −1.0 OR < −2.4)**	Left heel bone mineral density T-values: (−1.0) – (−2.4)	**0.010**	0.631	0.407	0.826
**Fasting glucose: 1**^**st**^**, 4**^**th**^**vs 2**^**nd**^**, 3**^**rd**^**quartile (<4.20 mmol/L OR >6.40 mmol/L)**	Fasting glucose is within normal range: 4.20 – 6.40 mmol/L	0.471	0.890	0.620	1.228
**Total serum cholesterol (<5.0 mmol/L)**	Total serum cholesterol: 5.0+ mmol/L	0.354	0.845	0.558	1.240
**Bilirubin in serum by sex-specific median (men = <11.0 µmol/L; women = <9.0 µmol/L)**	Bilirubin in serum: men = 11.0+ µmol/L; women = 9.0+ µmol/L	0.103	0.758	0.519	1.054
**Albumin in serum: 1**^**st**^**, 4**^**th**^**vs 2**^**nd**^**, 3**^**rd**^**quartile (<40 g/L OR >48 g/L)**	Albumin in serum: <40 g/L OR >48 g/L	0.464	1.144	0.770	1.603
**Iron in serum by sex-specific median (men = <14 µmol/L; women = <12 µmol/L)**	Iron in serum: men = 14+ µmol/L; women = 12+ µmol/L	0.225	0.800	0.547	1.160
**Unsaturated Iron Binding Capacity: 1**^**st**^**, 4**^**th**^**vs 2**^**nd**^**, 3**^**rd**^**quartile (<26 µmol/L OR >59 µmol/L)**	UIBC is within normal range: 26–59 µmol/L	0.626	0.844	0.344	1.726
**Folates in serum by median (<=18.1 nmol/L)**	Folates in serum: >18.1 nmol/L	**0.017**	0.502	0.245	0.816
**Erythrocytes: 1**^**st**^**, 4**^**th**^**vs 2**^**nd**^**, 3**^**rd**^quartile (< 9.0 *10e12/L OR >15.0 *10e12/L)	Erythrocytes: < 9.0 *10e12/L OR >15.0 *10e12/L	0.549	1.152	0.703	2.028
**Basophils by median (0.02+ %)**	Basophils*:* 0.02+ %	0.621	1.102	0.748	1.709
**Self-rated health (poor, satisfactory, good)**	Self-rated health: very good, excellent	0.556	0.892	0.558	1.349
**Self-rated health compared to age-peers (worse or equal)**	Self-rated health is better compared to age-peers	0.084	0.727	0.485	1.063
**Functional ability (self-rated mobility and independence are both less than excellent)**	Self-rated mobility and/or independence are excellent	0.440	0.861	0.556	1.324
**Mini Mental State Examination score by median (< 23)**	Mini Mental State Examination score: 23+	0.208	0.792	0.498	1.099
**Self-rated nutritional status (mildly or severely malnourished)**	Self-rated nutritional status: well nourished	**0.028**	0.599	0.334	0.881
**Mild or heavy depression (Yes)**	Not suffering from depression	0.080	0.745	0.512	1.045
**Number of medicaments taken daily (5+)**	Number of medicaments taken daily: 0–4	**0.044**	0.685	0.430	0.946
**Number of hospital admissions in the past year (2 or more)**	One or no hospital admissions in the past year	**0.020**	0.612	0.372	0.900
**Experiencing an acute illness in past 3 months (No)**	Experiencing an acute illness in past 3 months	0.074	0.701	0.437	1.047
**Family history of hypertension (No)**	No family history of hypertension	0.948	1.014	0.627	1.662
**Family history of diabetes (No)**	Have a family history of diabetes	0.277	0.760	0.442	1.311
**Smoking status (smoker or ex-smoker)**	Smoker or ex-smoker	0.656	1.086	0.724	1.613
**For the question: “Do you think that smoking is your health-risk behavior?” (Answer: “Yes“)**	Does not think that smoking is his/her health risk behavior	0.097	0.468	0.137	1.189
**Using denture (No)**	Does not use denture	0.348	1.220	0.780	1.976
**Number of vitamin supplements daily taken (men: 0–2; women: 0–1)**	Number of vitamin supplements daily taken: men: 0–2; women: 0–1	0.570	1.110	0.770	1.639
**Regularly taking supplementary vitamin B complex (No)**	Regularly taking supplementary vitamin B complex	**0.017**	0.533	0.292	0.966
**Regularly taking supplementary magnesium (No)**	Regularly taking supplementary magnesium	0.731	0.911	0.471	1.618
**Drinking beer (rare or never)**	Drinking beer at least once a week	0.426	0.772	0.356	1.380
**Age of the oldest living sibling (<80)**	Age of the oldest living sibling is 80+ years	**0.035**	0.560	0.289	0.897
**Mother's age at death (<80)**	Mother's age at death is 80+ years	**0.049**	0.738	0.506	0.983
**RTL**	RTL	0.491	1.052	0.910	1.250

Except RTL, all predictor variables are binary (0, 1) and the referent value “0” is described in parentheses. Significant p-values (p<0.05) are marked in bold.

### 3.3 Joint analyses of SNP interactions and health indicators

To determine whether the genetic interactions and the tested health status indicators have an independent effect on survival, significant SNP-SNP interaction models were tested along with the nine significant health-related variables (Supplementary Table 4). Out of 14 interaction models that were significant in the previous step, ten remain significant even in a bootstrapped model containing health variables, and they are presented in Table 4 in a decreasing order of interaction p-values. Five of the interactions that remained significant after the inclusion of health indicators in the analysis included one of the *CDKN2B* SNPs: the interactions of *TP53* rs1042522 with both *CDKN2B* rs4977756 and rs1333049 (p=0.002 and p=0.013, respectively), the interactions of two *FOXO3* SNPs, rs4946935 and rs12206094, with *CDKN2B* rs1333049 (p=0.035 and p=0.044, respectively); as well as the interaction between *LINC02227* rs2149954 and *CDKN2B* rs1333049 (p=0.015). The interactions between the two SNPs near *TERC*, rs16847897 and rs12696304, and *SH2B3* rs3184504 (p=0.007 and p=0.049, respectively) also remained significant after the addition of health-related parameters, along with the interactions between *IL6* rs1800795 and *GHRH*R rs2267723 (p=0.005), *LINC02227* rs2149954 and *PARK7* rs225119 (p=0.016), and *FOXO3A* rs2802292 and *ERCC2* rs50871 (p=0.047). While most health-related variables remain significant in models that include genetic interactions, the variable reporting the number of medicaments taken loses significance in both *TERC* – *SH2B3* interaction models, and the maternal age at death is no longer a significant predictor of survival in both interaction models of *CDKN2B* and *FOXO3,* indicating that these genetic interactions likely impact survival through a phenotype that is covered by these health parameters*.* The number of hospital admissions also loses significance in both *CDKN2B* – *FOXO3* models, the *PARK7* – *LINC02227* model, and one of the *CDKN2B* – *TP53* models*,* while being only marginally significant in the other.

**Table 4:** The results of Cox regression analyses of the models combining significant genetic interactions and nine selected health-related variables.

***First gene***	***CDKN2B*****rs4977756**	***IL6*****rs1800795**	***TERC*****rs16847897**	***CDKN2B*****rs1333049**	***LINC02227 rs2149954***	***PARK7*****rs225119**	**FOXO3 rs4946935**	***FOXO3*****rs12206094**	***FOXO3*****rs2802292**	***TERC*****rs12696304**
***Second gene***	***TP53*****rs1042522**	***GHRHR*****rs2267723**	***SH2B3*****rs3184504**	***TP53*****rs1042522**	***CDKN2B rs1333049***	***LINC02227*****rs2149954**	**CDKN2B rs1333049**	***CDKN2B*****rs1333049**	***ERCC2*****rs50871**	***SH2B3*****rs3184504**
**Interaction p-value**	**0.002**	**0.005**	**0.007**	**0.013**	**0.015**	**0.016**	**0.035**	**0.044**	**0.047**	**0.049**
**Gender**	0.311	0.146	0.078	0.182	0.292	0.082	0.248	0.238	0.183	0.134
**Body weight by sex-specific 4**^**th**^**quartile**	**0.001**	**0.002**	**0.001**	**0.002**	**0.003**	**0.001**	**0.001**	**0.001**	**0.001**	**0.001**
**Left heel bone mineral density**	**0.004**	**0.008**	**0.007**	**0.002**	**0.001**	**0.005**	**0.003**	**0.006**	**0.002**	**0.004**
**Folates in serum by median**	**0.023**	**0.001**	**0.019**	**0.009**	**0.011**	**0.018**	**0.014**	**0.023**	**0.020**	**0.014**
**Self-rated nutritional status**	**0.003**	**0.008**	**0.025**	**0.003**	**0.007**	**0.018**	**0.023**	**0.019**	**0.007**	**0.014**
**Number of medicaments taken daily**	**0.030**	**0.019**	0.114	**0.039**	**0.013**	**0.040**	**0.017**	**0.017**	**0.034**	0.074
**Number of hospital admissions in the past year**	0.069	**0.014**	**0.037**	**0.050**	**0.040**	0.074	0.095	0.102	**0.039**	**0.037**
**Regularly taking supplementary vitamin B complex**	**0.001**	**0.001**	**0.001**	**0.001**	**0.001**	**0.003**	**0.002**	**0.002**	**0.006**	**0.001**
**Age of the oldest living sibling**	**0.005**	**0.003**	**0.003**	**0.005**	**0.005**	**0.001**	**0.003**	**0.001**	**0.004**	**0.003**
**Mother's age at death**	**0.029**	**0.017**	**0.018**	**0.036**	**0.049**	**0.029**	0.056	0.063	**0.031**	**0.034**

The table shows bootstrap-adjusted p-values of gender, the SNP-SNP interactions, and nine health-related variables that made up the models that were tested by Cox regression analysis. The values of health-related variables are described in Table 3. Significant p-values (p<0.05) are marked in bold.

## 4 Discussion

In this study, we tested the effect of SNPs associated with longevity in other studies on survival above 85 years of age in a sample of Croatian oldest-old individuals, both individually and in two-SNP interactions. Individually, none of the SNPs had a significant effect on survival in advanced old age. Considering the relatively small sample size, the effect of the individual SNPs was possibly too weak to be detected at this level. However, the predictive power of SNPs can be improved by combining multiple SNPs in a single model (Van Den Broeck et al., 2014), or by testing the interactions between them, as SNP-SNP interactions may be more informative about the target phenotype than a single SNP alone (Gerke et al., 2009). The use of this approach for genetic studies of human longevity was validated in a study by (Dato et al. (2018); Dato et al., 2018), who investigated SNP-SNP interactions impacting longevity in a sample of Danish origin, while focusing on SNPs from three candidate pathways connected to longevity – the IIS, DNA repair, and pro/antioxidant pathways. Their approach was different from the one presented in this paper, as they studied a larger SNP dataset on a much larger sample and used the tagging approach to prioritize SNPs inside the candidate genes. They also applied a multi-dimensional reduction analysis, which we did not do here. We investigated interactions between all the SNPs we had available for our sample. SNP-SNP interactions were not examined for pairs of SNPs in LD, as linkage between loci might also falsely indicate a higher value of interaction (Su et al., 2015). As we also excluded from the interaction analyses all SNPs that had a very low frequency of one of the genotypes (less than 10 carriers of a homozygous genotype) to avoid false-positive results, the final set for interaction analyses comprised 28 SNPs. Of the 359 different models we tested, 10 SNP-SNP interaction pairs were nominally significant predictors of survival beyond the age of 85 years.

### 4.1 CDKN2B – the link between pathways with implications for longevity

Half of the two-SNP interactions that had an impact on survival above 85 years of age had an SNP associated with the *CDKN2B* gene as one of the members of the interacting pair, indicating a key role this gene has in longevity and late-life survival. Intronic variants rs4977756 and rs1333049, previously associated with longevity phenotypes (Pinós et al., 2014, Fortney et al., 2015, Pilling et al., 2016), are located in the chromosomic 9p21.3 region between the genes *CDKN2A* and *CDKN2B,* and are predicted in the online database Open Targets Genetics to most likely impact the expression of these genes, with the strongest evidence existing for *CDKN2B* (Ghoussaini et al., 2021). *CDKN2B* is a tumour suppressor gene that has been strongly associated with risk for coronary heart disease (Helgadottir et al., 2007, Burton et al., 2007, McPherson et al., 2007). It encodes protein p15^INK4B^, an inhibitor of cyclin-dependent kinases 4 and 6 that stops cell cycle progression in response to regulatory signals (Park and Lee, 2003), and has an important role in cell cycle regulation and senescence (McPherson et al., 2007). The expression of p15^INK4B^ is strongly induced by transforming growth factor-β (TGF-β) (Hannon and Beach, 1994), causing G1-phase cell cycle arrest. The genomic region around *CDKN2B* that spans across the two SNPs from this study also encodes a long non-coding RNA, *ANRIL*, that acts in *cis* via epigenetic mechanisms to silence the p15^INK4B^ expression and increase proliferation while slowing down the entry of cells into senescence (Kotake et al., 2011, Yap et al., 2010, Pasmant et al., 2011). Next to *CDKN2B* is the gene *CDKN2A* which encodes protein variants p16^INK4A^ and p14^ARF^ in two different reading frames (Pasmant et al., 2011). While p16^INK4A^ works similarly to p15^INK4B^ as a cell cycle inhibitor, p14^ARF^, on the other hand, acts by activating the p53 tumour suppressor pathway (Gil and Peters, 2006) by inhibiting protein MDM2, the key effector for degradation of p53 (Lohrum et al., 2000).

#### 4.1.1 Interactions within the cell cycle control network

In the current study, both SNPs associated with the *CDKN2B* gene interacted significantly with *TP53* rs1042522 to affect survival in the population above 85 years. This variant is a missense mutation causing substitution of arginine (Arg) with proline (Pro) at codon 72 of p53, a key tumour suppressor that blocks cell cycle progression (Lane, 1992, Lavin and Gueven, 2006) and promotes apoptosis in conditions of cellular stress (Shadyab et al., 2017). Under normal conditions, it is present in cells at low levels, but rapidly undergoes stabilising posttranslational modifications and activation in response to stimuli (Lavin and Gueven, 2006, Caspari, 2000). The effect of the Arg72Pro substitution is functional, with the proline variant having a reduced apoptotic response compared to the arginine (Marin et al., 2000, Dumont et al., 2003). This variant has also been reported to impact longevity and survival in the oldest-old age group (Van Heemst et al., 2005, Groß et al., 2014). As the potential effect on the expression of *CDKN2A*, and therefore p14^ARF^, has been reported for at least one of the SNPs in the *CDKN2A/B* region, the link between them and the *TP53* rs1042522 that we see in our study could be the via the p14^ARF^/MDM2/p53 axis, and the stabilizing effect p14^ARF^ has on p53. Furthermore, a study by Leeper et al. (2013) found that *CDKN2B* knockdown in human arterial smooth muscle cells resulted in increased expression of p53. They also performed protein microarray analysis of factors related to the p53 signalling and apoptotic pathways, and found that MDM2 protein, ahead of p53 itself, was among the top targets of proteins that are regulated (Leeper et al., 2013). This shows that *CDKN2B* may regulate p53 activity by mediating its degradation via MDM2.

#### 4.1.2 Interplay between cell cycle control and insulin signalling

The complex cellular network of insulin signalling and its downstream effects represents probably the best-studied system with implications for longevity. The *FOXO* genes are a group of transcription factors that act downstream of insulin and insulin-like growth factor receptors (Martins et al., 2016). As the most important transcriptional effectors of the IIS, FOXOs are activated by metabolic stress and lack of nutrients (Dong et al., 2008, Eijkelenboom and Burgering, 2013). Insulin or IGF-1 trigger a phosphatidylinositol 3-kinase/protein kinase B (PI3K-AKT) cascade, causing the serine/threonine kinase AKT to phosphorylate FOXO, which is followed by exclusion of FOXO from the nucleus and silencing of the genes targeted by FOXO (Biggs et al., 1999, Brunet et al., 1999, Webb and Brunet, 2014). The genes downstream of FOXO are involved in cellular quality control, proteostasis and autophagy (Mammucari et al., 2007, Kikis et al., 2010). *FOXO3* is a gene whose implication in longevity is well established, and the association of SNPs near *FOXO3* with longevity has been confirmed in diverse populations (Willcox et al., 2008, Anselmi et al., 2009, Flachsbart et al., 2009, Soerensen et al., 2010, Bao et al., 2014, Broer et al., 2015, Zeng et al., 2010). Four variants, whose association with the *FOXO3* gene are also implicated in the Open Targets Genetics database (Ghoussaini et al., 2021), had a significant interaction with *CDKN2B* rs1333049 that contributed to survival above 85 years in our oldest-old sample. This is perhaps not surprising, as *FOXO3* is upstream of the *CDKN2B* gene, acting as a regulator of *CDKN2B* expression (Hornsveld et al., 2018). Additionally, one study showed that FOXOs might be key interacting partners for SMAD transcription factors through which TGF-β pathway activates the *CDKN2B* gene expression (Gomis et al., 2006), which is what might explain the joint effect they have on longevity.

#### 4.1.3 Genetic risk factors for cardiovascular diseases

Another significant interaction partner of *CDKN2B* rs1333049 was variant rs2149954, located in the 5q33.3 genomic region, and close to the long intergenic non-coding RNA 2227 (*LINC02227*). This variant was first mentioned in a paper by Deelen et al. (2014) reporting results of GWAS on longevity as a novel locus associated with survival beyond 90 years of age (Deelen et al., 2014). Prior to this, variants in LD with this SNP have been associated with blood pressure and hypertension (Ehret et al., 2011, Wain et al., 2011). Zeng et al. (2016) confirmed the association with longevity in their GWAS on Han Chinese population (Zeng et al., 2016), which was replicated in another study (Liu et al., 2021). There is no data on the functional impact of this variant in the online databases, but the minor allele of rs2149954 was found to be protective against heart attack and heart failure, and was related with increased physical functioning in the long-lived individuals (Nygaard et al., 2017). Shadyab et al. (2017) found that seven SNPs in LD with rs2149954 impacted the chances of survival to age 85, which was explained by an increased risk of coronary heart disease connected to the one of the alleles (Shadyab et al., 2017). As a connection between genetic variants and CVD risk has been reported for both 5q33.3 region of rs2149954 and 9p21.3 region of *CDKN2B* rs1333049, the significant interaction of these SNPs for survival beyond the age of 85 might have something to do with modulating this risk.

*LINC02227* rs2149954 was also significant in interaction with rs225119, an intronic variant associated to the *PARK1* gene. *PARK7* encodes Parkinsonism associated deglycase, also known as DJ-1, an evolutionary conserved enzyme with a cysteine residue that serves as a catalytic nucleophile (Wilson et al., 2003) and a domain that shares a significant homology with a bacterial heat-shock protein (Wei et al., 2007). The cysteine residue is easily oxidised and has been reported to mitigate oxidative stress by serving as a scavenger for reactive oxygen species (ROS) (Clements et al., 2006, Chen et al., 2010, Billia et al., 2013, Shi et al., 2015). DJ-1 has been shown to affect cell survival to some degree by modulating PTEN/PI3K/Akt signalling cascade (Kim et al., 2005) and by altering p53 activity (Shinbo et al., 2005). Dato et al. (2018) have found the interaction of *PARK7* rs225119 with *MRE11A* rs533984 and *GHSR* rs572169 to be associated with longevity (Dato et al., 2018). The connection between *PARK1* and *LINC02227* is not very clear, but perhaps the antioxidative effect of *PARK7* works synergistically with the CVD-protective effect of *LINC02227* rs2149954 to influence survival chances.

### 4.2 Connection between CVD genetic risk factors and SNPs influencing telomere length

Intronic variants rs16847897, rs12696304 and rs3772190 are located on chromosome 3 near the *TERC* gene. Encoding the RNA component of the ribonucleoprotein telomerase, an enzyme that serves as a template and elongates telomeric DNA (Blackburn and Collins, 2011; Zhang et al., 2012), the *TERC* gene is an important component for telomere maintenance. It is an enzyme that is not expressed in most human cells (Blackburn et al., 2015), but is expressed in stem cells (Wright et al., 1996, Collins and Mitchell, 2002) and often in cancer cells (Hahn et al., 1999). All three of the SNPs have been associated with leukocyte telomere length (Codd et al., 2010, Soerensen et al., 2012, Shen et al., 2011), a phenotype that has been proposed as a marker of biological age (Sanders and Newman, 2013, Lohman et al., 2021) and associated with age-related diseases (Panossian et al., 2003, Aviv, 2012, Rossiello et al., 2022, Jeanclos et al., 1998). Functional analysis, however, links all three of these SNPs to changes in expression levels of another gene, *ACTRT3* (Ghoussaini et al., 2021), whose function has yet to be characterized. The missing link between these SNPs and *TERC* in databases reporting the results of functional analyses could be due to the fact that the product of the *TERC* gene is of RNA nature, and isn’t covered in analyses of protein expression. rs16847897, rs12696304 and rs3772190 all interacted with missense rs3184504 in the *SH2B3* gene in a way that significantly affected survival above 85 years of age*,* with the most significant interaction being between rs16847897 and rs3184504. The *SH2B3* gene encodes SH2B adaptor protein 3 (also known as LNK, lymphocyte adaptor protein), a protein whose main role is negative regulation of inflammatory cytokine signalling and haematopoiesis (Tong et al., 2005, Devallire and Charreau, 2011). rs3184504 is a common missense variant resulting in substitution of tryptophan (Trp) with arginine (Arg) at amino-acid 262, and is predicted to have the strongest impact on the SH2B3 (LNK) itself, disrupting its subcellular localisation and functioning (Dale and Madhur, 2016). This variant has been associated with exceptional human longevity and parental age (Fortney et al., 2015, Pilling et al., 2016). It is also is a top association signal for hypertension in GWAS (Ehret et al., 2011, Levy et al., 2009), and has been linked to cardiovascular and autoimmune disorders (Devallire and Charreau, 2011, Laroumanie et al., 2018). As telomere length and *SH2B3* both impact the chances for developing cardiovascular disease (CVD), the connection between the *TERC* and *SH2B3* genes could lie in disease pathophysiology. Since the incidence of cardiovascular pathologies increases with age (Lye and Donnellan, 2000), with an estimated prevalence of CVD among people over the age of 80 being 82% (Yazdanyar and Newman, 2009), it would make sense for the interactions of these two genes to have an impact on survival in this age group via a joint effect of protective variants in CVD evasion.

### 4.3 Interactions within broader IIS network

#### 4.3.1 Interplay of SNPs associated with obesity and IIS

The intronic variant rs50871 is located in the *ERCC2* gene, a gene encoding a DNA helicase that is an essential subunit of a complex transcription factor known as the general transcription factor 2 H (TFIIH) in charge of basal transcription, and is also involved in transcription-coupled nucleotide excision repair (NER) (Coin et al., 1998, De Boer and Hoeijmakers, 2000, Keriel et al., 2002, Benhamou and Sarasin, 2002). Functional analyses, however, report that rs50871 impacts the expression of *KLC3* gene (Ghoussaini et al., 2021) encoding kinesin light chain 3, a subunit of the molecular motor protein kinesin. While not much is known about the specific role of *KLC3*, apart from its ability to attach to mitochondria and its involvement in sperm tail formation, this gene has been associated with the development of Alzheimer’s disease and obesity metrics (Charisis et al., 2023). While Dato et al. (2018) report that rs50871 had a significant effect on longevity in interaction with *TP53* rs2078486 (Dato et al., 2018), in our study, rs50871 interacted significantly with the *FOXO3* gene rs2802292, which has also been associated with longevity (Flachsbart et al., 2009), especially in men (Willcox et al., 2008, Anselmi et al., 2009, Bao et al., 2014). With FOXO3 being a main connecting link to the IIS, and rs50871 causing changes to the expression of the protein related to obesity, it is possible that the SNP-SNP interaction between these two variants is significant due to the obesity-related changes in insulin signaling (Blackburn and Collins, 2011; Zhang et al., 2012).

#### 4.3.2 Interaction with genes from the growth hormone-IIS axis

Intronic variant rs2267723 is reported to influence the splicing of *GHRHR*, a gene that encodes growth hormone-releasing hormone receptor. A part of growth hormone/insulin-like growth factor 1/insulin signalling axis, this receptor, located in the pituitary gland on the membrane of somatotropic cells, binds growth hormone-releasing hormone which causes synthesis and secretion of growth hormone (GH) (Mayo et al., 2000). rs2267723 interacted significantly with rs1800795, an intronic variant that has previously been associated with the *IL6* gene, but is located closest to the *STEAP1B* gene. While there is evidence it influences the expression of both genes, the effect on *STEAP1B* is much stronger (Ghoussaini et al., 2021). Not much is known of the biological functions of *STEAP1B* genes, apart from their metalloreductase activity and their role in iron and copper homeostasis, (Ohgami et al., 2006, Xu et al., 2022) so it is difficult to assume how the variant associated with this gene works together with *GHRHR* rs2267723 to impact survival of the oldest-olds*.* Perhaps their interaction is dependent on the effect of rs1800795 on *IL6*, a cytokine with both pro- and anti-inflammatory properties (Minciullo et al., 2016) that has previously been associated with longevity (Christiansen et al., 2004, Albani et al., 2009, Revelas et al., 2018), and can influence insulin signalling and glucose metabolism (Kim et al., 2008).

### 4.4 Genetic interactions and health status indicators

The key factor for benefitting from the extra years of life attained on account of beneficial genetic background is good health (Beard et al., 2016). Existence of disease and its onset, functional status and frailty are all indicators of physiological changes that can precede death (Crimmins and Beltrán-Sánchez, 2011), and can be useful as variables for predicting survival. In this study, we tested the dataset of health-related parameters for our oldest-old sample as predictors of survival in advanced old age, both independently and with the significant genetic factors. Of the 33 tested variables, a subset of nine had an effect on survival in a model without genetic factors. These were maternal and fraternal longevity, nourishment status, weight, bone density, folates, number of medicaments taken, taking of B-complex supplements and number of hospital stays in the year prior to taking the survey. For most of these, the category within the variable related to better survival was an expected one, except that higher chances of survival were found for participants who had osteopenia. However, this is not entirely surprising, as osteopenia is a common trait amongst the oldest-old, thus representing normal ageing (Ginsburg et al., 2001, Škarić-Jurić and Rudan, 1997, Raisz and Seeman, 2001). In the joint models of genetic and health-related factors, most of the health-related variables remained significant, proving that the selected health-related traits can indeed robustly and independently of genetic factors predict chances of survival for the oldest-old population. Interestingly, loss of significance of the variable describing the number of medicaments taken in combination with *TERC* and *SH2B3* indicates that this interaction influences a phenotype that is also covered by these health parameters. Perhaps, it might have a role in mediating the number of chronic age-related conditions which are most often the cause of polypharmacy (Kurczewska-Michalak et al., 2021). Furthermore, loss of significance of maternal age at death in models with *CDKN2B* – *FOXO3* interactions indicates that the phenotype targeted by this genetic interaction has to do with familial longevity and lifespan.

Only four of the genetic interactions stopped being significant with the addition of the health-related variables, probably due to the introduction of variables that impacted the same phenotype as them. The interactions that remained significant, however, highlight the importance of cell cycle control and its interplay with IIS, the two main pathways with implications for longevity, but also indicate the vital role that modulators of cardiovascular risk and proteins with antioxidative effect have in determining survival chances. Furthermore, these findings imply that health status and health-related indicators are not the sole determinants of the dynamics of the ageing process.

### 4.5 Strengths and limitations of the study

Principal limitations of this study are the relatively small sample size and limited number of genotyped genetic loci, which both lead to the findings that were only nominally significant. Those limitations were partially compensated by generating bootstrap-adjusted results that present a more accurately predicted p-value. This study does, however, focus on SNPs with a strong previous association in studies with more power, and emphasizes the SNP-SNP interactions. By using the two-SNP-interaction method, it was possible to elucidate an effect that might not be detected otherwise. In addition, the analysis of SNP-SNP interactions is a valid method for finding significant genetic contributors in studies with low power, even though the statistical strength of the interaction analysis would also benefit from a larger sample size. Finally, it is, to our knowledge, the first study of genetic makeup contributing to survival of the oldest-olds in the Croatian population, a population otherwise underrepresented in genetic studies. Therefore, our study presents these initial results, but the obtained associations should be replicated in a population with a different genetic background, and a much larger sample size.

## 5 Conclusion

In conclusion, this study explored the effect of SNP-SNP interactions on survival above 85 years of age in a sample of Croatian oldest-olds. By focusing on genetic interaction between the longevity-associated variants rather than the individual SNPs, it was possible to identify pathways that contribute to survival in advanced old age. We identified a nominally significant interaction between SNPs in *CDKN2B* and *FOXO3*, *TP53* and *LINC02227* SNPs, as well as several other combinations that remain significant even when tested together with health status indicators. This shows that the interplay between genetic variants in different genes may affect survival in a manner that is not explained by biomarkers of health status and should be further explored in studies with larger sample sizes.

## Funding acknowledgements

Financial support was provided by 10.13039/501100004488Croatian Science Foundation (grants IP-01-2018-2497, HECUBA project; and DOK-2018-09-8382) and the 10.13039/501100000780European Union (ERC, ElucidAge,101041331). Views and opinions expressed are however those of the author(s) only and do not necessarily reflect those of the European Union or the European Research Council Executive Agency. Neither the European Union nor the granting authority can be held responsible for them. Neither of the funding sources had any involvement in study design; collection, analysis and interpretation of data; in the writing of the report or the decision to submit the article for publication.

## Declarations of interest

None.

The sample collection and the research described here were approved by the Ethics Committee of Institute for Anthropological Research (Zagreb, Croatia) and performed following all institutional guidelines. Ethical approvals obtained on March 4th 2006 (130–981/06) and 22nd November 2018 (20180518).

## CRediT authorship contribution statement

**Maja Šetinc:** Writing – review & editing, Writing – original draft, Visualization, Validation, Investigation, Formal analysis, Data curation. **Željka Celinšćak:** Writing – review & editing, Visualization, Validation, Investigation, Data curation. **Luka Bočkor:** Writing – review & editing, Supervision, Resources, Investigation. **Matea Zajc Petranović:** Writing – review & editing, Data curation. **Anita Stojanović Marković:** Writing – review & editing, Validation. **Marijana Peričić Salihović:** Writing – review & editing, Supervision. **Joris Deelen:** Writing – review & editing, Supervision, Funding acquisition, Conceptualization. **Tatjana Škarić-Jurić:** Writing – review & editing, Validation, Supervision, Project administration, Funding acquisition, Data curation, Conceptualization.

## Data Availability

Fully anonymised dataset of genetic data used in this study is publicly available on Zenodo repository (DOI: 10.5281/zenodo.7421684). Data on health-related parameters is available upon request.
